# Microglia-Derived NLRP3 Activation Mediates the Pressor Effect of Prorenin in the Rostral Ventrolateral Medulla of Stress-Induced Hypertensive Rats

**DOI:** 10.1007/s12264-020-00484-9

**Published:** 2020-04-03

**Authors:** Li Hu, Shutian Zhang, Kokwin Ooi, Xuehai Wu, Jiaxiang Wu, Jian Cai, Yinggang Sun, Jijiang Wang, Danian Zhu, Fuxue Chen, Chunmei Xia

**Affiliations:** 1 Laboratory of Neuropharmacology and Neurotoxicology, Shanghai Key Laboratory of Bio-Energy Crops, College of Life Science, Shanghai University, Shanghai, 200444 China; 2 Department of Physiology and Pathophysiology, Basic Medicine College, Fudan University, Shanghai, 200032 China; 3 Department of Neurosurgery, Huashan Hospital, Fudan University, Shanghai, 200040 China; 4 Department of Neurology, Renji Hospital, Shanghai Jiaotong University, Shanghai, 200240 China; 5 Department of Cardiovascular Diseases, Xinhua Hospital Affiliated to Shanghai Jiao Tong University School of Medicine, Shanghai, 200025 China

**Keywords:** Stress, NLRP3, Prorenin, Microglia, Hypertension

## Abstract

Increased microglial activation and neuroinflammation within autonomic brain regions such as the rostral ventrolateral medulla (RVLM) have been implicated in stress-induced hypertension (SIH). Prorenin, a member of the brain renin-angiotensin system (RAS), can directly activate microglia. The present study aimed to investigate the effects of prorenin on microglial activation in the RVLM of SIH rats. Rats were subjected to intermittent electric foot-shocks plus noise, this stress was administered for 2 h twice daily for 15 consecutive days, and mean arterial pressure (MAP) and renal sympathetic nerve activity (RSNA) were monitored. The results showed that MAP and RSNA were augmented, and this paralleled increased pro-inflammatory phenotype (M1) switching. Prorenin and its receptor (PRR) expression and the NLR family pyrin domain containing 3 (NLRP3) activation were increased in RVLM of SIH rats. In addition, PLX5622 (a microglial depletion agent), MCC950 (a NLRP3 inhibitor), and/or PRO20 (a (Pro)renin receptor antagonist) had antihypertensive effects in the rats. The NLRP3 expression in the RVLM was decreased in SIH rats treated with PLX5622. Mito-tracker staining showed translocation of NLRP3 from mitochondria to the cytoplasm in prorenin-stimulated microglia. Prorenin increased the ROS-triggering M1 phenotype-switching and NLRP3 activation, while MCC950 decreased the M1 polarization. In conclusion, upregulated prorenin in the RVLM may be involved in the pathogenesis of SIH, mediated by activation of the microglia-derived NLRP3 inflammasome. The link between prorenin and NLRP3 in microglia provides insights for the treatment of stress-related hypertension.

**Electronic supplementary material:**

The online version of this article (10.1007/s12264-020-00484-9) contains supplementary material, which is available to authorized users.

## Introduction

Evidence shows that chronic psychosocial stress is directly linked to the development of hypertension, cardiovascular disease, and stroke [1]. The activation of various neurogenic pathways, such as stimulation of the sympathetic nervous system (SNS), mediates not only short-term increases in blood pressure (BP), but also the elevation of chronic BP in response to specific stresses [2]. The sympathetic outflow is controlled by key cerebral nuclei and neural circuits in the central nervous system (CNS), predominantly the rostral ventrolateral medulla (RVLM) [3–5], the nucleus tractus solitarius, and the hypothalamic paraventricular nucleus (PVN), all of which are associated with the activities of the autonomic nervous system [2].

It is well established that increased SNS outflow and enhanced renin-angiotensin system (RAS) activity are common features of hypertension and various pathological settings that predispose individuals to hypertension. In an animal experiment, it has been reported that chronic foot-shock stress upregulates the expression of the RAS components in the CNS and the circulatory system, thereafter increases vasopressin, oxidative stress, and stress hormone levels, which are involved in the development of hypertension [1]. More recently, hypertension has been recognized as an immune condition and evidence suggests that reciprocal communication between the RAS, SNS, and immune systems plays a role in the establishment of hypertension [4, 5]. Increased SNS activity plays the roles of altering immune system responses in pathophysiological settings. Moreover, microglia, the resident central immune cells, also contribute to the increased SNS activity in rodents [6]. However, the cause-effect relationship between central inflammatory responses and the centrally-triggered sympathetic drive remains elusive.

Microglia maintain normal neuronal physiology and homeostasis in the CNS in their resting or immuno-surveillant stages [7]. There are M1/pro-inflammatory and M2/immune-regulatory microglial phenotypes. Our previous study showed that the centrally administered anti-inflammatory agent minocycline significantly alleviates M1 microglia activation and hypertension in SIH rats [6]. The main component of RAS, angiotensin II (Ang II) itself, is a powerful inducer of increased pressure. It also triggers microglial activation and boosts the production of pro-inflammatory cytokines [2] and reactive oxygen species (ROS) through mitochondrial damage, which subsequently contributes to the activation of sympathetic nerve activity in hypertension development [8–10]. It has been confirmed that the Ang II/AT1 receptor axis or prorenin directly activates microglia into the M1 immunophenotype. Ang II triggers the immune system *via* shifting the microglia from the M2 to the M1 phenotype by binding to the Ang II receptor type 1 (AT1) [9].

Prorenin as the precursor of renin can directly stimulate microglial activation and pro-inflammatory cytokine production, apart from initiating the RAS cascade, and this may contribute to activation of the pro-inflammatory phenotype (M1) [11–14]. It has been reported that microinjection of human prorenin and activation of the (pro)renin receptor (PRR) in the PVN contribute to the increased sympathetic nerve activity in rats [15]. A PRR agonist can bind both renin and prorenin, which leads to the non-proteolytic activation of prorenin and local Ang II formation [16]. In addition, it has been noted that while the PRR-(pro)renin complex plays its roles independent of Ang II, it also actives downstream signal transduction pathways such as the NF-κB and Wnt signaling pathway [11, 17]. Our previous *in vitro* study showed that prorenin induces the disintegration of mitochondria and M1 phenotype switching *via* ROS-overproduction in microglia [18], while its roles in BP regulation warrant further investigation.

The NLR family pyrin domain containing 3 (NLRP3) inflammasomes are multi-protein complexes that comprise the NLRP3 protein, the adaptor molecule apoptosis-associated speck-like protein containing a CARD (caspase recruitment domain) (ASC), and cysteine protease caspase-1 components [19]. NLRP3 inflammasomes are responsible for the maturation of pro-inflammatory cytokines such as interleukin-1β (IL-1β) and IL-18 involved in host defense and cellular stress responses [20, 21]. NLRP3 inflammasomes are activated by many mechanisms, such as K^+^ efflux, endoplasmic reticulum stress, intracellular Ca^2+^ and ROS. ROS might activate NLRP3 inflammasomes both as a trigger and an effector, resulting in pathological processes [22]. Zhou *et al.* first reported that the accumulation of damaged mitochondria increases the activation of NLRP3 inflammasomes [23]. NLRP3 was originally thought to be a cytosolic receptor, but it translocates to mitochondria in hypoxic settings [24]. Whether prorenin activates NLRP3 *via* triggering ROS and its translocation profile in the RVLM of SIH rats remain unknown.

Here, we investigated the mechanisms involved in the local effect of prorenin on microglial activation in SIH rats. We demonstrated that stress induced prorenin and PRR upregulation in the RVLM, which triggered microglial activation. We then confirmed that the prorenin-NLRP3 inflammatory signaling in microglia mediated the SNS activation during stress-induced pathology.

## Materials and Methods

### Drugs and Reagents

N-acetylcysteine (NAC; A7250), dimethyl sulfoxide, 4′,6-diamidino-2-phenylindole, and anti-β-actin (A1978) were from Sigma-Aldrich (St. Louis, MO). Recombinant human prorenin was from Abcam (ab93266; Cambridge, MA) and MCC950 (sc-505904) was from Santa Cruz Biotechnology (Santa Cruz, CA). The ROS fluorescent probe-DCFH-DA kit (S0033) and caspase 1 activity assay kit (C1101) were from the Beyotime Institute of Biotechnology (Nanjing, China). The following antibodies were used: sheep monoclonal antiserum against prorenin/renin (GTX7967, Gene Tex, San Antonio, TX); rabbit polyclonal antiserum against PRR (bs-7691R, Bioss, Beijing, China); rabbit polyclonal antiserum against caspase-1 (D7F10); rabbit anti-ASC monoclonal antibody (#13833), anti-CD86 (#91882), and anti-CD206 (#91992) were from Cell Signaling Technology (Beverly, MA); mouse monoclonal antiserum against CD11b/c (OX42, ab1211), mouse monoclonal to IL-6 (ab9324), iNOS (ab15323), Arg (ab60176), Iba-1 (ab153696), GFAP (ab7260), PGP 9.5 (ab10410), and rabbit polyclonal antiserum against NLRP3 (ab214185) were from Abcam. Donkey anti-sheep IgG H&L Alexa Fluor 555 (ab150178), goat anti-rabbit IgG H&L Alexa Fluor 594 (ab150080), and goat anti-mouse IgG H&L Alexa Fluor 488 (ab150117) were from Abcam. MCC950 was from Adipogen Corp. (San Diego, CA). PLX5622 was from Plexxikon (Berkeley, CA).

### Generation of the PRR Peptide Ligand

PRO20 was synthesized by Sangon Biotech (Shanghai) Co., Ltd. (Shanghai, China) as previously described [25].

### Animals

Adult Sprague-Dawley rats (male, 8 weeks old, 250–300 g) were purchased from the Animal Laboratory Center of Fudan University. Altogether, 140 animals were used in this study, with a morality rate of 5% due to intolerance of brain surgery, or death during stress. All experimental procedures were approved by Fudan University Animal Care Committee and conformed to the guidelines of the Institutional Ethics Committee; all efforts were made to minimize the number of animals used and their suffering. The rats were housed under a 12-h light/dark cycle in a temperature-controlled room at 24 °C with standard food and tap water *ad libitum*. They were divided into the following groups (6 rats each): (i) normotensive (Ctrl); (ii) stress-induced hypertensive (SIH); (iii) SIH + aCSF (artificial cerebrospinal fluid); (iv) SIH + MCC950 (a selective NLRP3 inhibitor), (v) Ctrl + MCC950; (vi) SIH + PRO20 (a newly-developed PRR antagonist, 200 μmol/L); and (vii) Ctrl + PRO20. PLX5622 was used to eliminate microglia in SIH rats. MCC950 was released into the cisterna magna at 0.15 µL/h from an osmotic mini-pump to maintain a total final concentration of 0.5 µmol/L in the brain [10]. PRO20 or MCC950 was delivered by osmotic minipump once daily for 1 week (from stress day 8 to day 15). PLX5622 was formulated in standard chow by the Department of Laboratory Animal Science, Fudan University, at 1200 ppm, and administered for 7 consecutive days (days 8–15 of stress). Molecular studies were replicated at least 3 times.

In preliminary experiments, we assessed the M1 polarization in the rats. The results showed that the inflammatory microglial marker CD86 and their secreted proteins (iNOS, Arg, and IL-6) did not differ between control and drug treatment (MCC950 or PRO20) groups, implying that the drugs themselves did not have pro- or anti-inflammatory effects in normal rats (Fig. S1). In accord with the guidelines of the Institutional Ethics Committee to minimize the number of animals used and their suffering, further molecular experiments were conducted in the Ctrl, SIH, SIH + vehicle (aSCF), SIH + MCC950, and SIH + PRO20 groups.

SIH was induced as described in our previous publications [4, 5]. Briefly, rats were placed in a cage (22 cm × 22 cm × 28 cm) and received intermittent electric shocks (35–75 V, 0.5 ms in duration) every 2–30 s randomly controlled by a computer. Meanwhile, noise (range, 88–98 dB) produced by a buzzer was given as the conditioned stimulus [6]. The rats were subjected to stress for 2 h twice daily for 15 consecutive days. The control group underwent sham stress. Systolic blood pressure (SBP) was recorded in conscious rats using the tail-cuff method. SBP measurements were repeated three times and the average value was taken.

### General Procedures for Acute Experiments

As in our previous study [6], rats were anesthetized with sodium pentobarbitone (50 mg/kg) intraperitoneally (i.p.). A catheter was placed in the femoral vein. BP was measured *via* a femoral artery cannula connected to a pressure transducer and a polygraph (PowerLab system, AD Instruments, Bella Vista, NSW, Australia). The HR was automatically derived from the phasic arterial BP wave. Body temperature was maintained at 37 °C by a heating pad.

### Implantation of Intracisternal Osmotic Minipump

The procedures were performed as described in our previous study [6]. Briefly, the rats were anesthetized with pentobarbital sodium (50 mg/kg, i.p.). After a midline dorsal neck incision, the dura was perforated with a 22-gauge steel needle and following cerebrospinal fluid leakage, a PE-5 catheter (Clay Adams, Sparks, MD) was advanced 5 mm into the cisterna magna and sealed to the dura with tissue glue. The outer end of the catheter was connected to a micro-osmotic minipump (Alzet 1007D, Durect Co., Cupertino, CA). The PRR antagonist (PRO20) or MCC950 was delivered by osmotic minipump. The rats received procaine penicillin injection (1,000 IU, i.m.) postoperatively. Rats that had progressive weight gain and were in normal physiological condition after the operation were used in subsequent experiments.

### Renal Sympathetic Nerve Activity (RSNA) Recording

We recorded RSNA when the general procedures for acute experiments were ready. Before a renal sympathetic nerve was exposed, the trachea was cannulated with polyethylene tubing and connected to a pneumotachograph (SAR-830/P, CWE Inc., Ardmore, PA) to maintain normal ventilation. Then, the head was fixed in a model 920 Kopf stereotaxic frame with bars and the body was placed in the lateral position on a heating pad. This approach is similar to that used by Huber and Basu [15]. Briefly, rats were anaesthetized as above and the left kidney was exposed through a retroperitoneal incision. A pair of silver recording electrodes was placed on the isolated left renal sympathetic nerve (Teflon 786500, A-M Systems Inc., Sequim, WA). Subsequently, the exposed nerve and the electrodes were secured with Kwik-Sil gel (World Precision Instruments) and a Grass P55C preamplifier was used to amplify and filter (bandwidth: 100–3,000 Hz) the nerve activity. The maximum activity occurred 1–2 min after the rat was overdosed by narcotic euthanasia. Baseline nerve activity was taken as the percentage of maximum after the noise level was subtracted; the background noise level was recorded 15–20 min after the rat was euthanized using the unit conversion of the PowerLab Chart system (AD Instruments).

### Primary Culture of Rat Microglial Cells and *In Vitro* Experimental Design

Primary cultures of microglial cells were prepared as previously described [26]. Briefly, the medulla oblongata covering the RVLM was removed from 1–2-day-old Sprague-Dawley rats after decapitation as we described previously [6]. The RVLM was identified according to the atlas of Watson and Paxinos [27]. Both sides of the RVLM (about 1.5- to 2.5-mm lateral to the midline and medial to the spinal trigeminal tract) were collected using micropunches with a 1-mm inner diameter burr. Next, Hanks balanced salt solution dissecting medium containing glucose, bovine serum albumin (BSA) and HEPES, as well as 0.025% trypsin was used to incubate the minced tissue at 37 °C for 20 min. Then, cells were plated at 3 × 10^5^ cells/cm^2^ in Dulbecco’s modified Eagle’s medium (DMEM) with GlutaMax and high glucose (4.5 g/L), supplemented with 10% fetal bovine serum, 0.1 mg/mL streptomycin and 100 U/mL penicillin in poly-*l*-lysine-coated 75 cm^2^ culture flasks. After 3 days, the culture medium was removed and replaced with fresh medium and kept at 37 °C under 95% O_2_/5% CO_2_. On day 9, the cells were re-suspended after centrifugation (150 × g for 10 min) [28]. Cell viability was evaluated by trypan blue exclusion (Fig. S2). The purity of cultured microglia was >95% when evaluated by flow cytometry.

For the *in vitro* experiments, microglia were divided into to 6 groups: (i) control (Ctrl, vehicle); (ii) prorenin (20 nmol/L for 24 h) treatment (PRO) [13, 18]; (iii) Ctrl + NAC (5 mmol/L); (iv) Ctrl + MCC950 (10 μmol/L); (v) PRO + NAC (5 mmol/L); and (vi) PRO + MCC950 (10 μmol/L). The concentrations of NAC [29] and MCC950 were determined as described previously [30]. We preliminarily investigated the effects of MCC950 and/or NAC on M1 polarization and the release of pro-inflammatory factors (IL-1β and TNF-α) from microglia in the control group. There results showed no significant differences between the vehicle (Ctrl) and drug-treatment (MCC950 and NAC) groups, which implied that the drugs themselves did not affect control-group microglia (Fig. S3). Therefore, further molecular experiments were mainly conducted in the Ctrl, PRO, PRO + NAC, and/or PRO + MCC950 groups.

### Flow Cytometry

In order to measure M1 and M2 polarization, primary culture of microglia was carried out as described previously [18, 31]. Microglia were harvested by re-suspension in cold phosphate-buffered saline (PBS) containing 0.5% BSA/0.05% NaN_3_, then incubated in 20% DMEM/F12 medium. M1 and M2 phenotype microglia were recognized using monoclonal antibodies specific for CD86-PE and antibodies specific for CD206-APC, respectively. For immunophenotypic analysis, the number of purified microglia was adjusted to 1 × 10^6^/mL. Primary microglia were incubated for 30 min with 10% fetal bovine serum, followed by incubation with the antibody mixtures for 30 min on ice. Microglia were washed and re-suspended in PBS twice, and then immediately assessed by flow cytometry (Becton Dickinson, Swindon, UK) and analyzed using Flowing software v2.5.1. Flow cytometry analysis of the expression profiles of CD86 and CD206 allowed us to distinguish M1 (CD86^+^) from M2 (CD206^+^) [32].

### Double Immunofluorescence Staining and Confocal Microscopic Imaging

Immunofluorescence was performed as described previously [5, 6], to determine the co-localization of NLRP3 with microglia CD11b/c (OX42), astrocytes (GFAP), and neurons (NeuN). Rats were anesthetized with 10% chloral hydrate and perfused through the left ventricle with 200 mL of 0.01 mol/L PBS (pH 7.4), followed by 200 mL of freshly-prepared 4% paraformaldehyde in 0.1 mol/L PB. RVLM sections were collected, post-fixed for 4 h, then placed in 20% and 30% sucrose at 4 °C to dehydrate overnight. Free-floating 30-μm coronal sections containing the RVLM were cut on a cryostat (Microm, Walldorf, Germany) [33]. The sections were washed in PBS and incubated with 0.3% Triton X-100 for 30 min followed by incubation with 5% horse serum for 1 h at 37 °C to block non-specific protein. The sections were incubated with the polyclonal antibodies CD86, CD206, NLRP3, CD11b/c, GFAP, and PGP9.5 overnight at 4 °C. We used goat anti-mouse IgG H&L Alexa Fluor 488, donkey anti-sheep IgG H&L Alexa Fluor 647, and donkey anti-rabbit IgG H&L Alexa Fluor 594 secondary antiserum. NLRP3 co-localization with mito-tracker was investigated *in vitro*. The fluorescent signals were monitored under a Fluorview FV300 laser scanning confocal microscope (Olympus, Tokyo, Japan); immunoreactivity manifested as specific green or red fluorescence.

### Western Blot Analysis

Total RVLM tissue (see Fig. S4 for location of micropunctures) from each rat was homogenized in lysis buffer with 1% NP40 and 1 mmol/L PMSF. Protein samples of the same amount from each rat were extracted from RVLM homogenates to analyze protein expression by western blot. In brief, the protein samples (20 μg each) were subjected to SDS/PAGE in 8%–12% gradient gel (Invitrogen, Carlsbad, CA) and transferred to PVDF membrane. Prorenin, PRR, NLRP3, ASC, caspase-1, pro-IL-1β, IL-1β, Iba-1, and M1 markers [CD86, IL-6, iNOS, and Arg] in the RVLM and/or cultured microglia were measured. This was followed by incubation with horseradish peroxidase-conjugated goat anti-rabbit IgG or goat anti-mouse IgG. The amount of protein was assessed by ECL detection reagents (WBKLS0050; Millipore) and the immunostaining band was visualized and quantitated by an automatic chemiluminescence image analysis system (Tanon-5200; Tanon Science & Technology, Shanghai, China). The data were normalized by developing the β-actin as loading control. The concentration of all the antibodies was 1:1000 except for β-actin (1:5000).

### RNA Extraction and Quantitative Real-Time Polymerase Chain Reaction (PCR)

Total RNA extraction reagent (TaKaRa, Dalian, China) was used to extract the total RNA from the RVLM. The mRNAs of prorenin, PRR, NLRP3, pro-Casp-1, ASC, IL-1β, TNF-α, IL-10, and TGF-β were analyzed by quantitative real-time PCR. Isolated mRNA was quantified by spectrophotometry and the 260/280 nm optical density ratio was calculated. The cDNA was synthesized using a high-capacity cDNA reverse transcription kit (Applied Biosystems, ABI). The relative quantification of gene expression was expressed as fold-change *via* normalization against β-actin by using the 2ΔΔCT method. The sequences of primers were designed using Primer Express 2.0 and are listed in Table S1.

### DCFH-DA Fluorescent Imaging to Analyze Intracellular ROS Production in Microglia

The ultrastructural mitochondrial morphology showed that prorenin-induced mitochondria had disorientation and cristae breakage (Fig. S5), so we measured ROS production in microglia. Microglia were seeded in a 6-well plate overnight and treated as previously described. DCFH-DA is a ROS-specific fluorescent probe, which we used to measure total intracellular ROS levels. The microglia were incubated with 10 μmol/L DCFH-DA at 37 °C in a dark room. After washing 3 times with serum-free medium, microglia were analyzed under a Fluorview FV300 laser scanning confocal microscope (Olympus), with an excitation wavelength of 488 nm and an emission wavelength of 525 nm.

### Statistical Analysis

Experimental data are expressed as the mean ± SEM (standard error of the mean). Student’s unpaired *t* test was used for experiments that contained two groups of samples. For comparative purposes, one-way or two-way analysis of variance with repeated measures was used to determine differences between groups. This was followed by the Tukey’s multiple range tests for *post hoc* assessment of individual means. *P* < 0.05 indicated that the differences were statistically significant. Statistical data were analyzed with GraphPad Prism 5 software.

## Results

### Prorenin and/or PRR Expression is Upregulated in the RVLM of SIH Rats

**Fig. 1:**
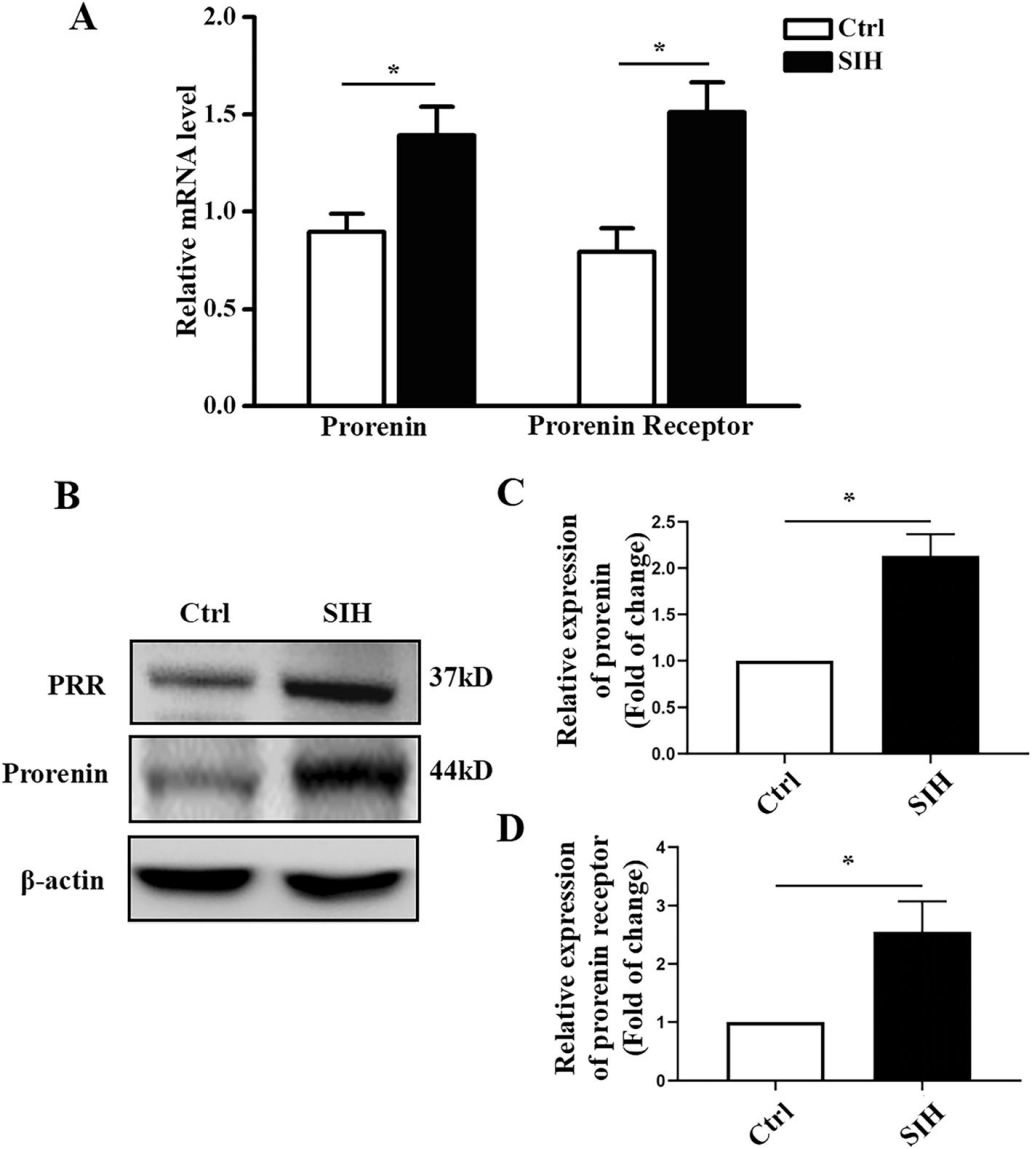
Expression of prorenin/PRR in the RVLM of rats. **A** qRT-PCR showing both prorenin and PRR are increased in the RVLM of SIH rats. **B** Representative western blots of prorenin and PRR in the RVLM of SIH rats. **C**, **D** Densitometric quantification showing prorenin and PRR are higher in the SIH group than in controls. Data are presented as the mean ± SEM. *P < 0.05 *vs* Ctrl, *n* = 6/group.

We measured the protein and mRNA levels of prorenin and/or PRR in the RVLM to determine whether stress caused their abnormal expression. We found that their mRNA levels were increased (Fig. 1A, *P* < 0.05) in the RVLM of SIH rats. Representative immunoblot bands of prorenin and/or PRR in the RVLM are shown in Fig. 1B. Western blotting analysis showed that the protein levels of prorenin and/or PRR were increased 2.1 and 2.5-fold respectively (Fig. 1C, D, *P* < 0.05) in SIH rats compared with controls.

### Mean Arterial Pressure (MAP) and RSNA are Augmented in SIH rats, and are Attenuated by the PRR Antagonist PRO20

**Fig. 2:**
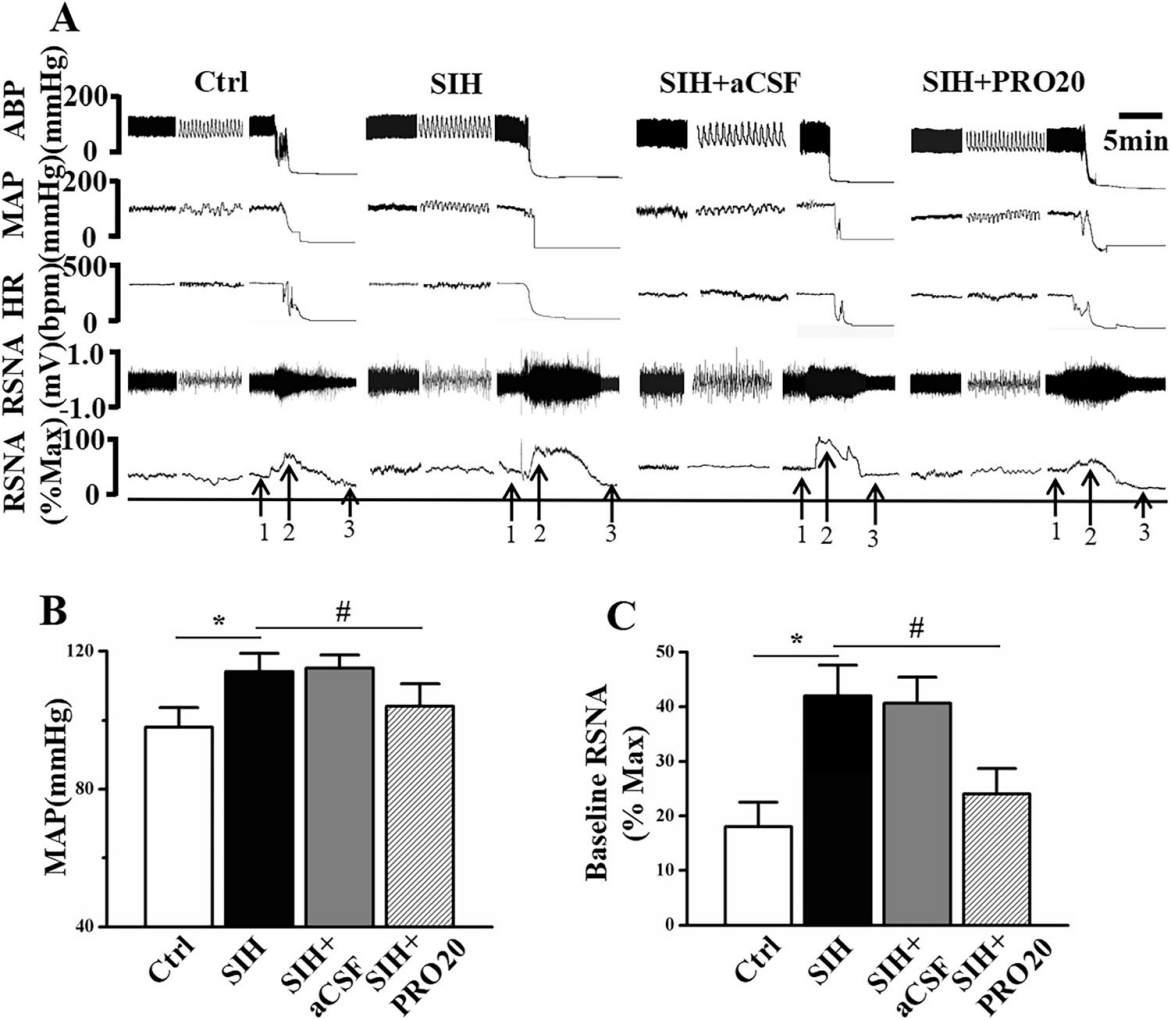
Effects of intracisternal infusion of PRO20 on the MAP and RSNA in SIH rats. **A** Representative original traces demonstrating the effect of PRO20 infusion on RSNA, MAP, and HR (1, basal RSNA; 2, maximum RSNA; 3, noise level). The maximum occurred 1–2 min after the rat was euthanized. Note that the maximum RSNA did not significantly differ among the 3 groups, while the SIH/SIH + aCSF rats had a greater basal RSNA level than control and/or PRO20-treated SIH rats, and intracisternal infusion PRO20 attenuated the MAP and baseline RSNA level of SIH rats. **B** Statistical data for MAP. **C** Statistical data for baseline nerve activity. Data represent the mean ± SEM; *n* = 6; *P < 0.05 *vs* Ctrl; ^#^*P* < 0.05 *vs* SIH. ABP, arterial blood pressure; MAP, mean arterial pressure; HR, heart rate; RSNA, renal sympathetic nerve activity; SIH, stress-induced hypertensive rats; aCSF, artificial cerebrospinal fluid.

Given the increased prorenin and PRR in the RVLM of SIH rats, we speculated that prorenin/PRR play a potential role in BP regulation. Next, we investigated whether MAP and RSNA are regulated by prorenin/PR. aCSF and the PRR antagonist PRO20 were infused into the cerebellomedullary cistern in SIH rats. Hemodynamic measurements and RSNA recordings showed that the arterial BP, mean MAP and RSNA were higher in the SIH group than in the control group, and these increases were attenuated by cerebellomedullary cistern infusion of PRO20 (*P* < 0.05, Fig. 2).

### M1/M2 Ratio and Pro-inflammatory Factors are Increased in the RVLM of SIH Rats, and are Attenuated by the PRR Antagonist PRO20

**Fig. 3:**
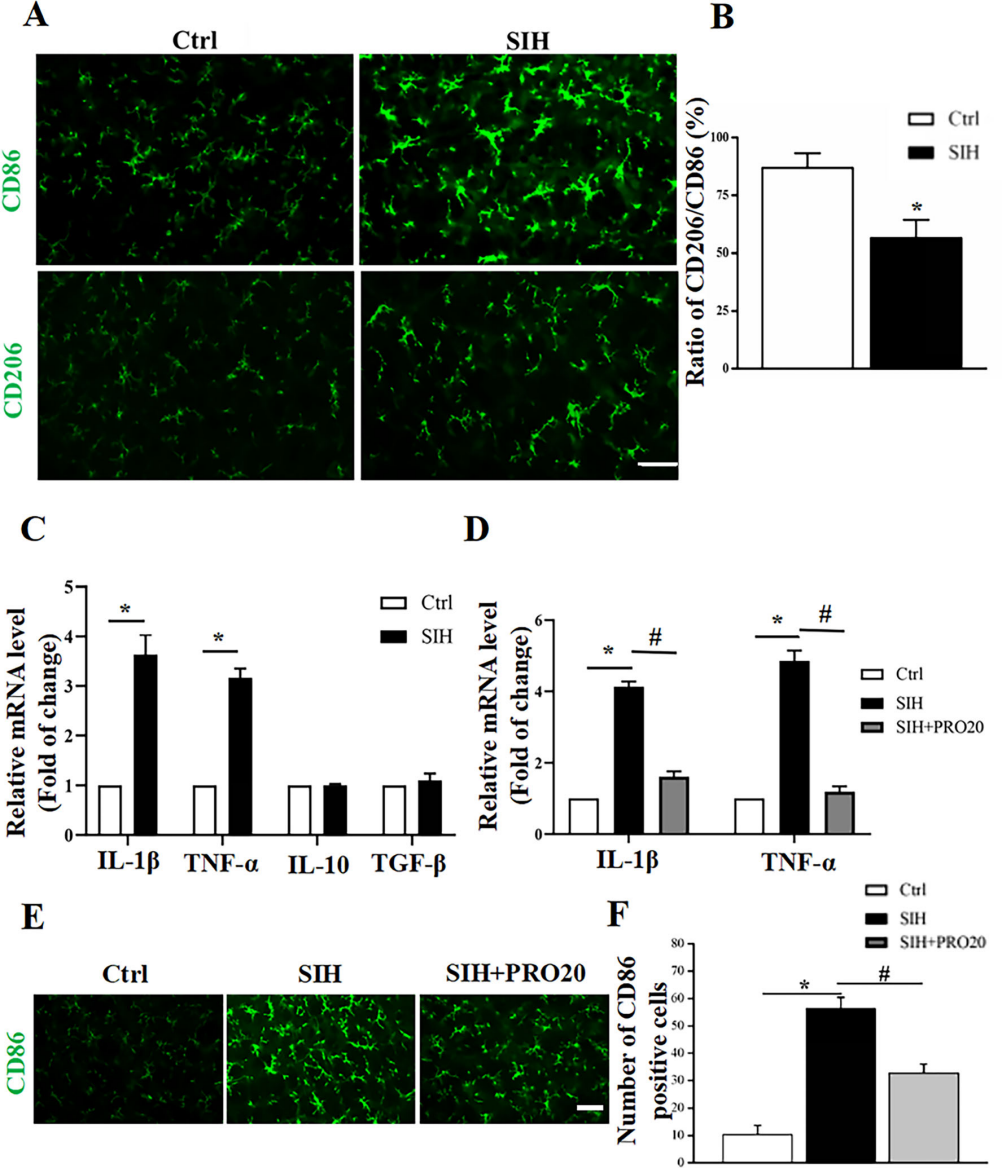
The ratio of M1/M2 polarization and release of pro-inflammatory/immune-regulatory factors in the RVLM of SIH rats. **A** Immunofluorescence detection of M1 and/or M2 phenotype microglia in the RVLM showed robust switching to M1 phenotype in SIH rats. **B** Relative ratio of M2 *versus* M1 phenotype microglia in the RVLM expressed as a percentage. **C**, **D** qRT-PCR showing the release of pro-inflammatory/immune-regulatory factors was increased in the RVLM of SIH rats. **E**, **F** The PRR antagonist, PRO20, decreased the M1 polarization in SIH rats. Data represent the mean ± SEM; *n* = 6; *P < 0.05 *vs* Ctrl; ^*#*^*P* < 0.05 *vs* SIH; scale bars, 200 μm in (**A**) and (**E**).

Activated microglia can be polarized to M1 and/or M2 phenotype [26], and the M1 phenotype indicates neuroinflammatory activity. The immunofluorescent staining showed that activated microglia were present in the RVLM, and we used the CD86 or CD206 antibody to identify the M1 or M2 phenotype of microglial activation (Fig. 3A). Immunofluorescence analysis showed that the ratio of M1 phenotypes was increased and that of M2 phenotypes was decreased in SIH rats (Fig. 3B, *P* < 0.05). Furthermore, neuroinflammation was identified by the release of pro-inflammatory and immune-regulatory factors (Fig. 3C). To determine the effect of PRO20 on neuroinflammation following SIH, we performed qRT-PCR and confirmed that the pro-inflammatory factor release was increased in SIH rats (Fig. 3C, *P* < 0.05), but further decreased in PRO20-treated SIH rats (Fig. 3D, *P* < 0.05). Immunofluorescence analysis confirmed that PRO20 treatment significantly reduced the M1 microglia, as shown by decreased CD86 staining (Fig. 3E, F, *P* < 0.05). These results indicated that PRO20 effectively inhibits the polarization of the M1 phenotype and reduces neuroinflammation in the RVLM of SIH rats.

### NLRP3 Inflammasome Expression is Increased in Both Microglia and Neurons in the RVLM of SIH Rats, and is Decreased by the PRR Antagonist PRO20

**Fig. 4:**
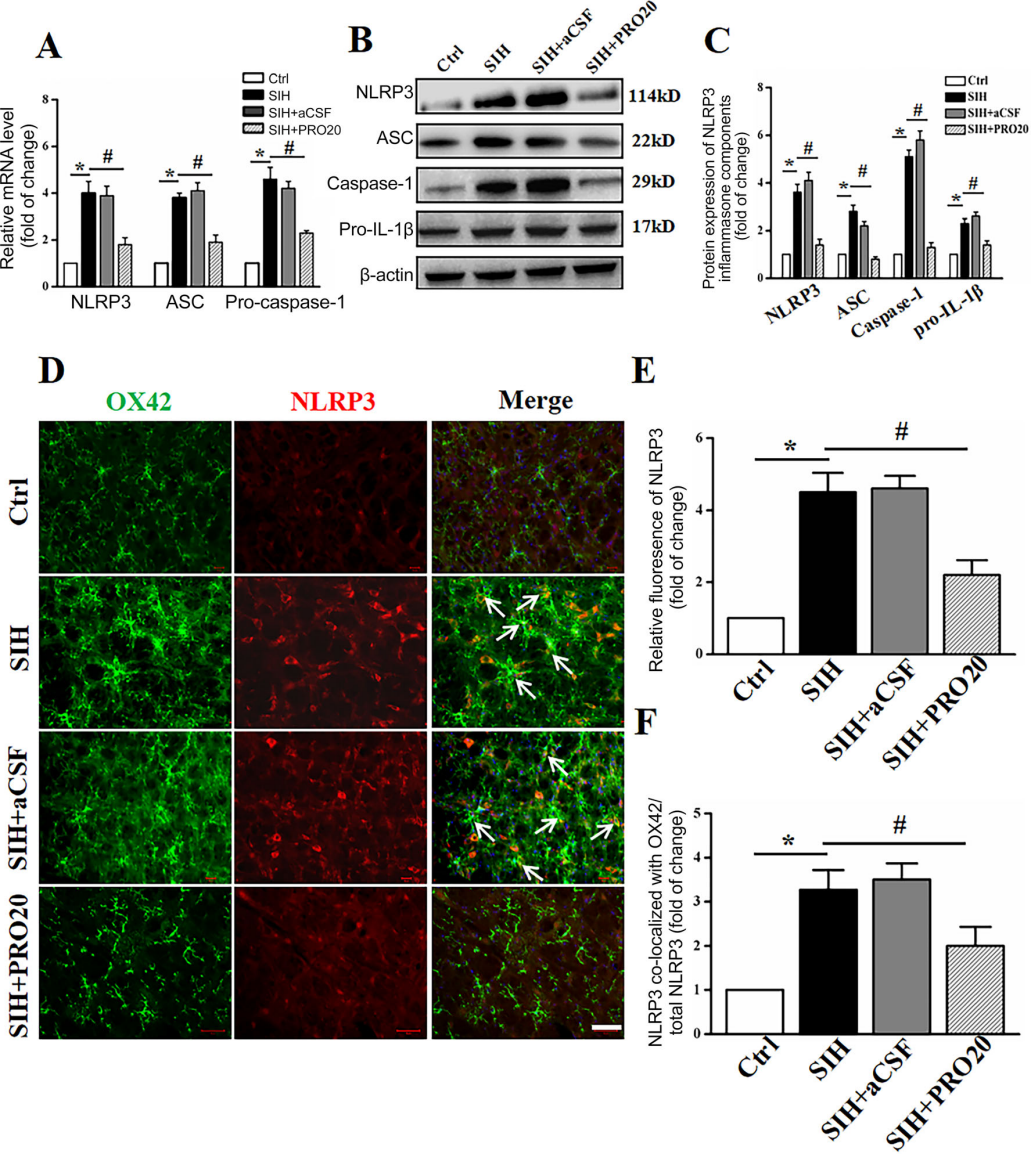

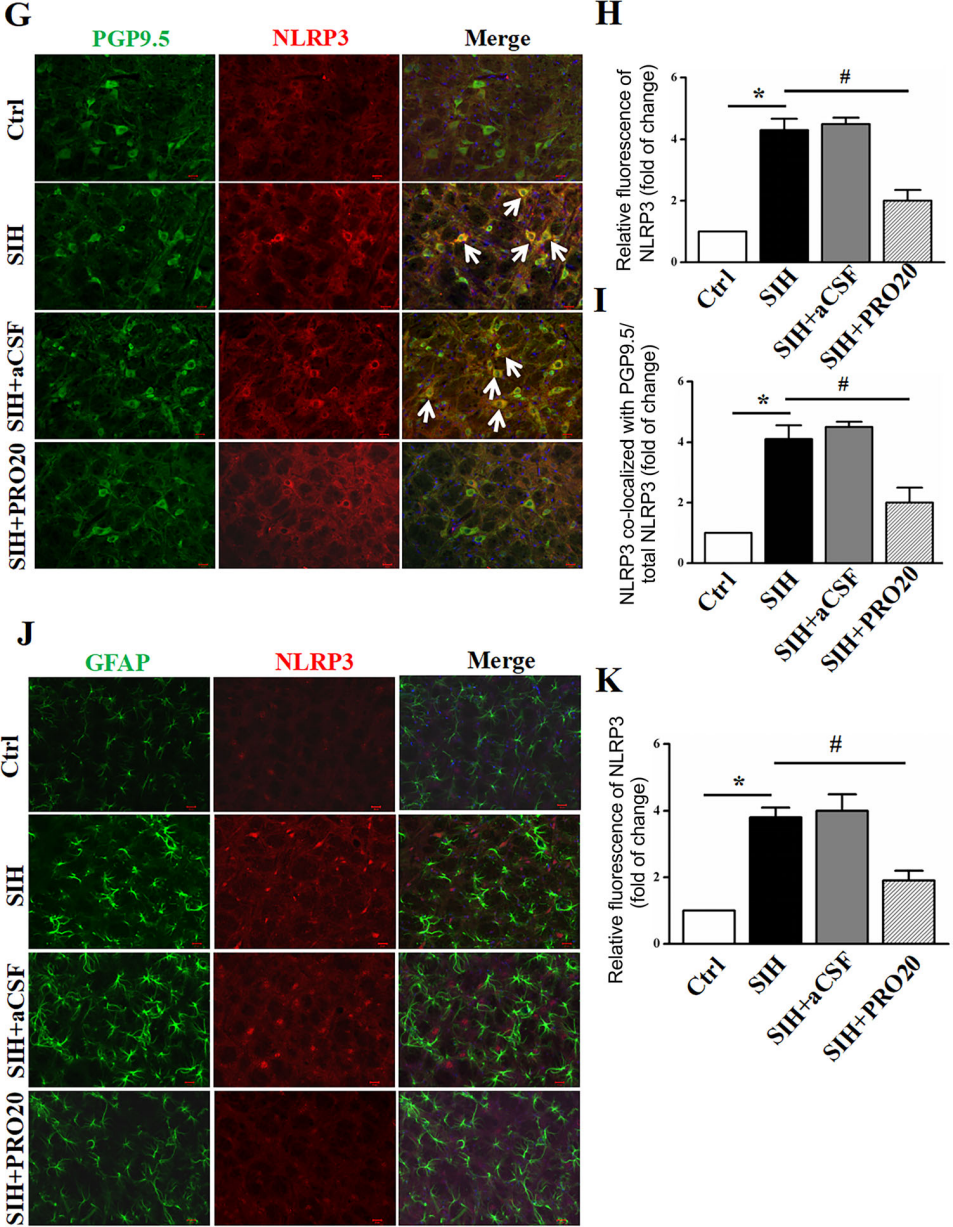
Expression of NLRP3 is downregulated by PRO20, a PRR antagonist. **A** Effect of PRO20 on the mRNA expression of NLRP3/ASC/caspase-1 using qRT-PCR analyses. **B**, **C** Representative immunoblots and quantitative analysis showing that NLRP3, ASC, caspase-1, and pro-IL-1β expression in the RVLM of SIH rats was upregulated, while PRO20 treatment downregulated their expression. **D** Representative images showing that NLRP3 (red) co-localized with the microglial marker (OX42). **E**, **F** Relative fluorescence intensity calculation of NLRP3-immnoreactivity with or without OX42-immnoreactivitiy in different groups. **G**–**I** Representative images and quantitative analysis showing co-localization of PGP 9.5 (neuronal marker, green) and NLRP3 (red) in the RVLM. **J**, **K** Representative images of double immunofluorescent staining for GFAP (astrocyte marker, green) and NLRP3 (red) and densitometric quantification of immunoreactivity in the RVLM. Notably, immunofluorescence staining analysis showed that NLRP3 mostly localized with microglia and neurons (arrows). Data represent the mean ± SEM; *n* = 6; *P < 0.05 *vs* Ctrl; ^#^*P* < 0.05 *vs* SIH; scale bars, 200 μm in (**D**), (**G**), and (**J**).

We first examined the role of PRO20 in NLRP3/ASC/caspase-1 mRNA expression using qRT-PCR analyses. Compared with those of SIH group, the mRNA levels of NLRP3, ASC, and caspase-1 p20 were significantly decreased in the PRO20-treated group (all *P* < 0.05, Fig. 4A). We then assessed the protein expression profiles of NLRP3 inflammasome components in the RVLM, and found that the NLRP3, ASC, and caspase-1 p20 protein levels (Fig. 4B, C) were significantly increased in SIH rats. Based on these results, double immunofluorescence staining was then performed to investigate which cell type expressed the upregulated NLRP3. We stained NLRP3 along with the microglia, neuron, and astrocyte markers, OX42, PGP9.5, and GFAP, respectively. The double immunofluorescent staining showed that NLRP3 mainly co-localized with microglia (Fig. 4D–F) and/or neurons (Fig. 4G–I) but was not co-expressed with the astrocyte marker GFAP (Fig. 4J, K). Immunofluorescence analysis confirmed that stress induced strong microglial activation and upregulation of NLRP3 expression in the RVLM (Fig. 4D–K, *P* < 0.05), while PRO20 significantly reduced the immunofluorescence density of NLRP3 both in microglia and neurons (Fig. 4F, I, *P* < 0.05). These results indicated that PRO20 effectively inhibits activation of the NLRP3 inflammasome in the RVLM of SIH rats.

### The Anti-hypertensive Effects of MCC950 Depend on the Presence of Microglia

**Fig. 5:**
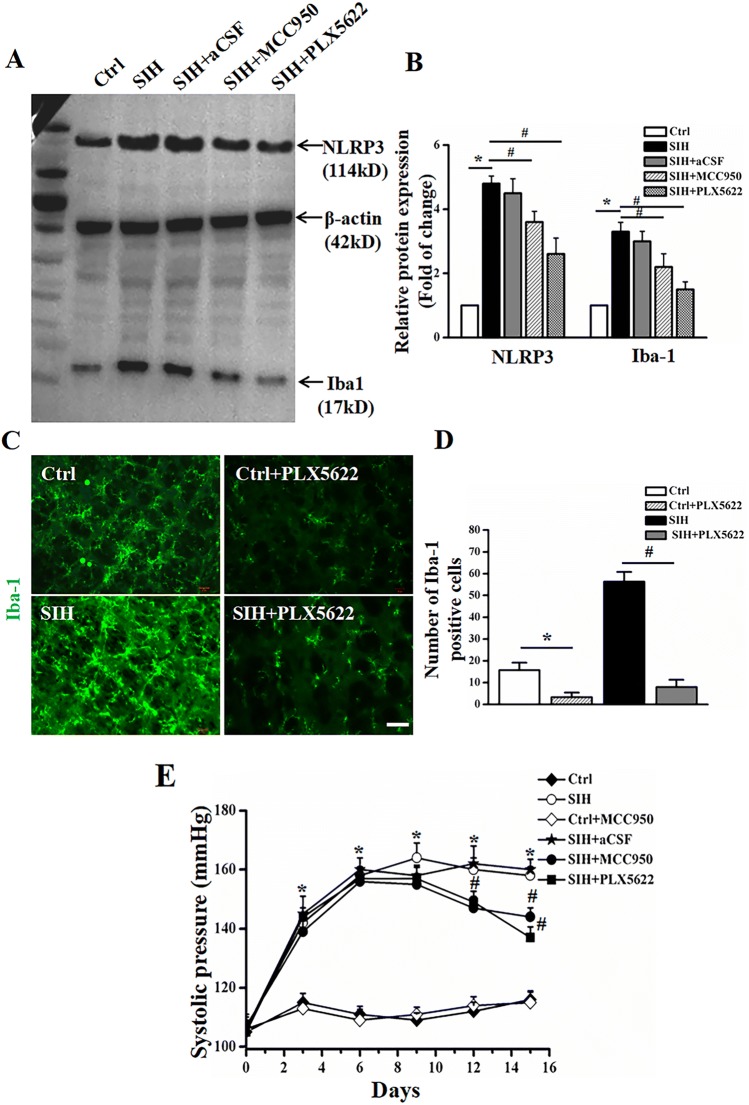
The NLRP3 inhibitor, MCC950, has an anti-hypertensive effect on SIH, which might be associated with microglia-derived NLRP3. **A**, **B** Representative immunoblots and densitometric analysis showing that NLRP3 expression is decreased in both MCC950 (NLRP3 inhibitor) and PLX5622 (CSF1R inhibitor)-treated SIH rats. **C**, **D** The efficiency of microglia elimination by PLX5622 evaluated by decreased microglia marker of Iba-1 using immunofluorescent staining. **E** SBP measurements showing that both MCC950 and PLX5622 had a depressor effect in SIH rats. Data represent the mean ± SEM; *n* = 6; *P < 0.05 *vs* Ctrl; ^*#*^*P* < 0.05 *vs* SIH.

Since microglia were the predominant cell type expressing NLRP3 following SIH, we further explored whether MCC950, the NLRP3 inflammasome inhibitor, had an anti-hypertensive effect by targeting microglia. We used a specific CSF1R inhibitor, PLX5622, to eliminate microglia as previously reported [34] after 7 successive days of SIH induction. The effect of microglial depletion by PLX5622 was identified using western blot analysis (Fig. 5A, B) and immunofluorescent staining for the microglia marker Iba-1 (Fig. 5C, D). Western blot analysis showed that PLX5622 treatment resulted in the elimination of ~85% of microglia and decreased NLRP3 expression in the RVLM of SIH rats (Fig. 5A, B). Immunofluorescent staining also confirmed that microglia were depleted by ~85%. Of note, we found that microglial elimination by PLX5622 attenuated the augmented blood pressure in SIH rats compared with the SIH + aCSF group. In addition, a depressor effect of MCC950 was observed in SIH rats (Fig. 5E, all *P* < 0.05). These results implied that microglia partially contribute to the anti-hypertensive effect of MCC950 in SIH rats.

### Prorenin Promotes the M2–to–M1 Polarization Transition *Via* NLRP3 Over-activation *In Vitro*

**Fig. 6:**
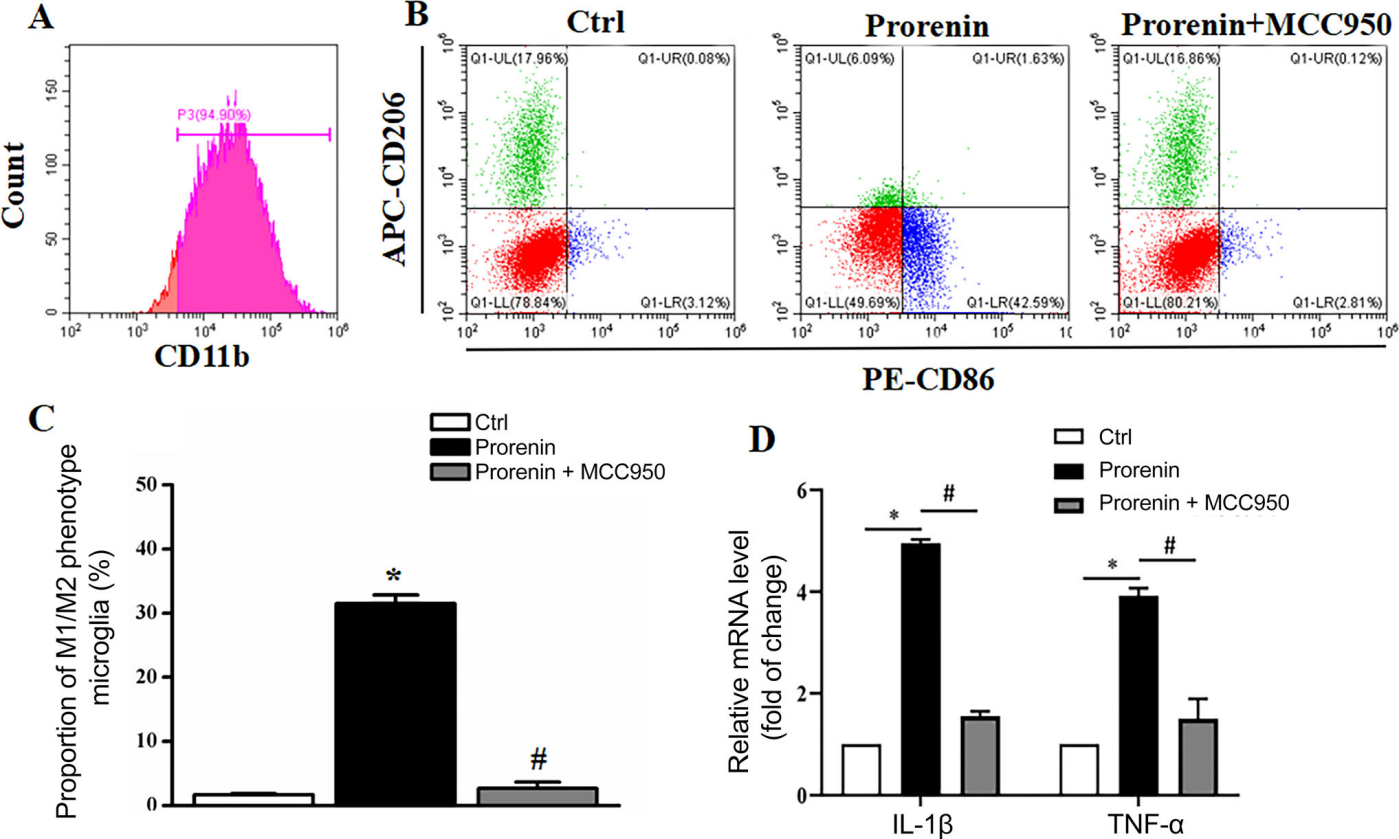
NLRP3 mediates M1 polarization in prorenin-treated microglia. **A** Analysis of the expression profile of CD11b by flow cytometry confirming that the purity of isolated primary microglia was > 94%. **B** Representative fluorescence-activated cell sorting plots showing the M1 (CD86+) and M2 (CD206+) phenotypes by flow cytometry in Ctrl, prorenin, and prorenin + MCC950-treated microglia. **C** Statistical data showing the proportions of M1/M2 phenotypes in Ctrl, prorenin, and prorenin + MCC950-treated microglia. **D** qRT-PCR analysis showing that the pro-inflammatory factors TNF-α and IL-β are increased in prorenin-treated microglia, while co-treatment with prorenin and MCC950, an NLRP3 inhibitor, decreases the release of TNF-α and IL-β. Data represent the mean ± SEM; *n* = 6; *P < 0.05 *vs* Ctrl; ^*#*^*P* < 0.05 *vs* prorenin.

To investigate whether prorenin regulates microglial polarization *via* NLRP3 activation, we incubated primary microglia cultured from rats with recombinant human prorenin (20 nmol/L) for 24 h. The purity of cultured microglia was > 94% when evaluated by flow cytometry (Fig. 6A). The M1 and M2 phenotypes were assessed by flow cytometry in the Ctrl, prorenin, and prorenin + MCC950 groups (Fig. 6B). There was a significant increase in the proportion of M1/M2 in prorenin-treated microglia (from 0.67% to 31.52%, *n* = 6, *P* < 0. 05), while co-treatment with prorenin and MCC950 reversed the M1/M2 proportion (from 31.52% to 3.97%, *n* = 6, *P* < 0.05, Fig. 6C). qRT-PCR analysis showed that prorenin in microglia increased the inflammatory factors TNF-α and IL-β (Fig. 6D, *P* < 0. 05). Conversely, interference with NLRP3 by its inhibitor MCC950 in microglia resulted in a decrease in M1-related inflammatory factors (Fig. 6D, *P* < 0. 05). These results indicated that suppressing NLRP3 activation reduces the ratio of M1 to M2 polarization and pro-inflammatory factor release, which implies that prorenin promotes M2–to–M1 polarization *via* NLRP3 over-activation *in vitro*.

### Prorenin Increases NLRP3 Activation, and This is Mediated by ROS Overproduction

**Fig. 7:**
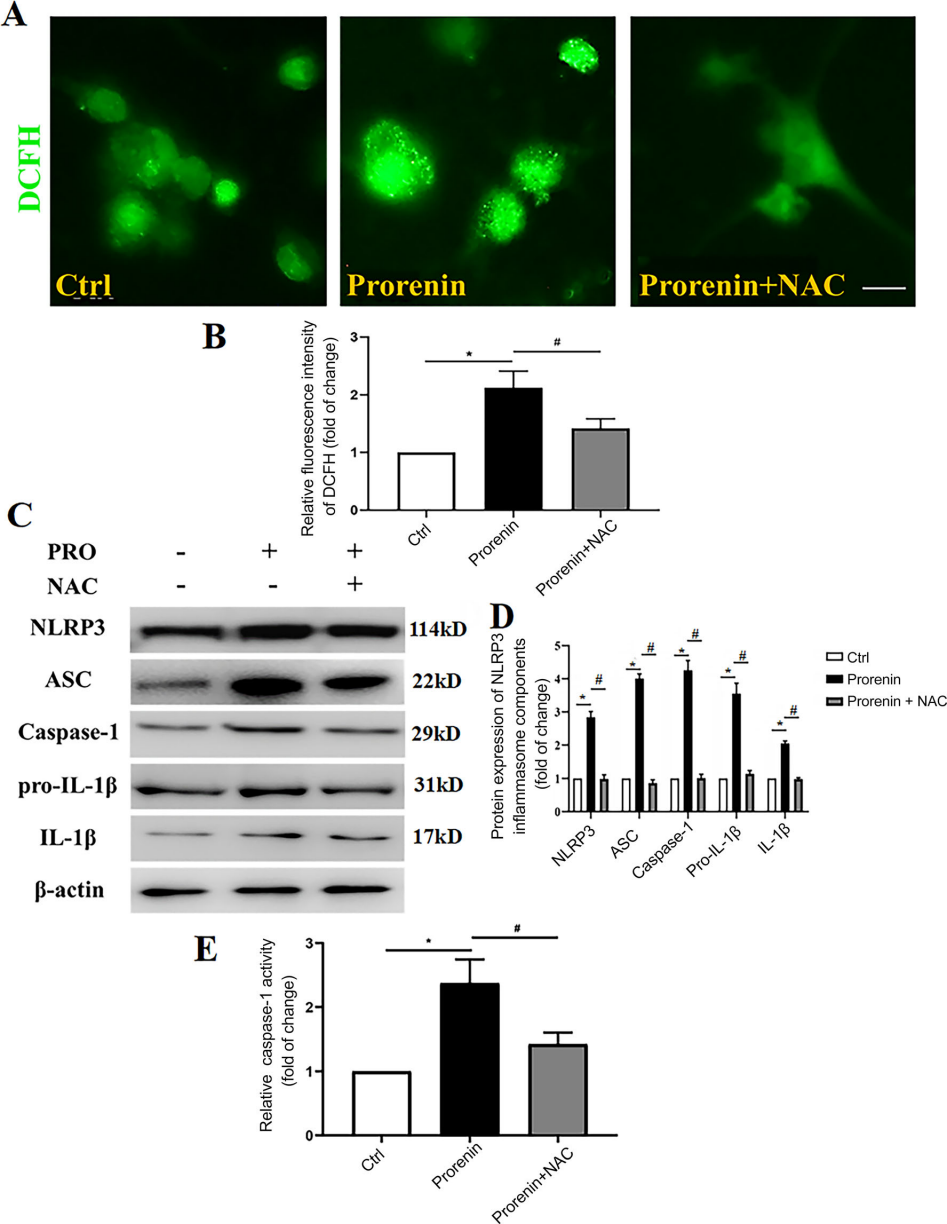
ROS mediate the prorenin-induced activation of the NLRP3 inflammasome. **A** Representative images of cytoplasmic ROS production in control, prorenin, and prorenin with NAC groups using DCFH-DA-associated fluorescence assays. **B** ROS quantification indicated by fluorescent intensity in the different groups. **C** Immunoblots of the components of the NLRP3 inflammasome (NLRP3, ASC, and caspase-1), pro-IL-1β, and its mature product IL-1β in prorenin with or without the ROS scavenger NAC. **D** Statistics for experiments as in **C**. **E** Caspase-1 activity. Data represent the mean ± SEM; *n* = 6, *P < 0. 05 *vs* Ctrl; ^#^*P* < 0. 05 *vs* prorenin, one-way ANOVA with unpaired *t* test; scale bar, 15 μm.

To investigate whether prorenin directly activates NLRP3 *via* oxidative stress, the ROS scavenger NAC was administered along with prorenin. Incubation of microglia with prorenin for 24 h elicited a significant increase in ROS prodnot the inflammasomes ofuction (*n* = 6, *P* < 0.05; Fig. 7A, B). The immunoblot results showed that components of the NLRP3 inflammasome (NLRP3, ASC, caspase-1), pro-IL-1β, and its mature product IL-1β were increased in the prorenin-treated group (*n* = 6, *P* < 0.05, Fig. 7C, D). Furthermore, there was increased inflammasome activation in prorenin-treated microglia compared with controls (*n* = 6, *P* < 0.05), as indicated by increased caspase-1 activity (Fig. 7E), and these effects were attenuated by co-treatment with the ROS scavenger NAC. This implied that prorenin increases NLRP3 activation, which is mediated by oxidative stress.

**Fig. 8:**
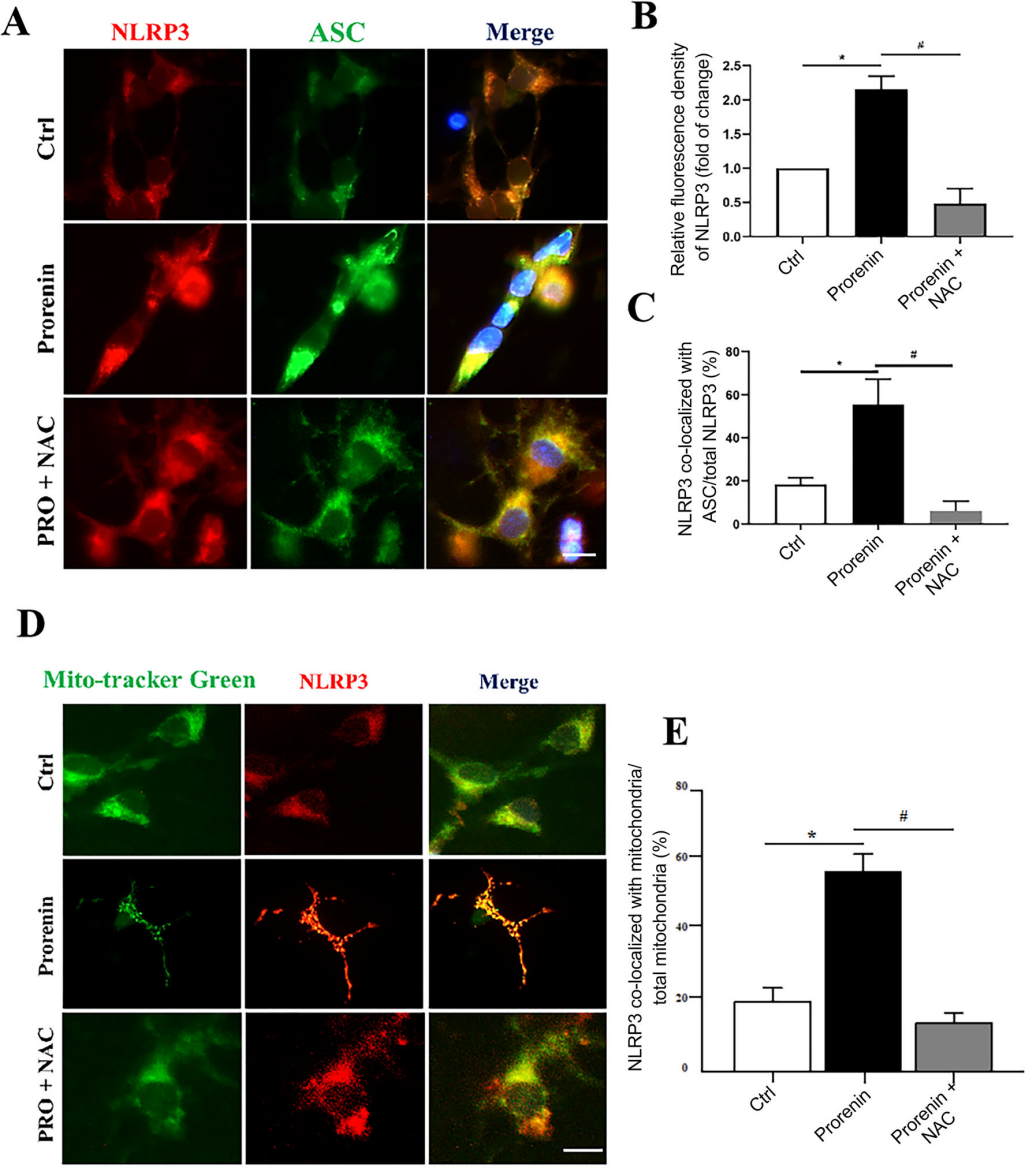
The increased NLRP3 inflammasome activation and NLRP3 translocation from cytoplasm to mitochondria are attenuated by co-treatment with prorenin and NAC. **A** Representative images of primary microglia co-stained with NLRP3 (red) and ACS (green). **B** Densitometric quantification of immunofluorescent staining for NLRP3 in different groups. **C** Co-localization of NLRP3 and ASC in control, prorenin, and prorenin plus NAC groups. **D** Representative images of primary microglia stained with NLRP3 (red) and mito-tracker (green). **E** Percentage co-localization of NLRP3 imunoreactivity with mitochondria. Data represent the mean ± SEM; *n* = 6; *P < 0.05 *vs* Ctrl; ^#^*P* < 0.05 *vs* prorenin, one-way ANOVA with unpaired t test; scale bars, 15 μm in **A** and **D**.

Immunofluorescent staining showed that the co-localization of NLRP3 and ASC components was increased in the prorenin-treated group (*n* = 6, *P* < 0.05, Fig. 8A–C), which was another profile of inflammasome activation. Co-staining for mito-tracker and NLRP3 showed much more NLRP3 translocation from the rest of cytoplasm to mitochondria, which meant that NLRP3 was activated to some extent (*n* = 6, *P* < 0.05, Fig. 8D, E), while the increased co-localization and translocation were attenuated by co-treatment with prorenin and NAC, which resulted in decreased ROS. The above results showed that prorenin increases ROS-mediated M1 phenotype-switching and NLRP3 activation in microglia *in vitro*.

## Discussion

The main purpose of the present study was to study the roles of prorenin in cardiovascular regulation by the RVLM, and further to identify the mechanisms underlying the pressor effect of prorenin. To this end, we measured the expression of endogenous prorenin and NLRP3 complexes in the RVLM of SIH rats. The roles of prorenin in the pathogenesis of SIH were identified using the centrally-administered PRR inhibitor PRO20 [35]. The effect of NLRP3 on BP was investigated using the selective NLRP3 inhibitor MCC950, which specifically inhibits the activation of NLRP3 but not the inflammasomes of AIM2, NLRP4, or NLRP1 [36]. However, with *in vivo* studies it has been difficult to reconcile whether these effects are associated with microglia. Accordingly, we used the microglial depletion agent PLX5622 to address this question as reported in the literature [37]. Furthermore, the effect of ROS on triggering NLRP3 activation was elucidated using the ROS scavenger NAC [38] in prorenin-treated microglia *in vitro*. We used intracisternal infusion to chronically administer pharmacological agents in order to assess their effects as in similar studies [6, 39, 40]. Our major findings were the following: SIH rats manifested increased BP and RSNA, implying increased sympathetic overactivity consistent with stress-induced neurogenic hypertension; intra-RVLM prorenin and PRR were upregulated, with increased M1 phenotype-switching and NLRP3 inflammasome activation in the RVLM of SIH rats. The microglia-depleting agent PLX5622, the NLRP3 inhibitor MCC950, or the PRR antagonist PRO20 had an antihypertensive effect in SIH rats. NLRP3 in the RVLM was decreased when rats were treated with PLX5622. Furthermore, NLRP3 with mito-tacker staining showed much more NLRP3 translocation from mitochondria to the cytoplasm in prorenin-stimulated microglia *in vitro*. Prorenin increased ROS-triggered M1 phenotype-switching and NLRP3 activation, while MCC950 decreased M1 polarization in prorenin-challenged microglia. We concluded that increased intra-RVLM prorenin might be involved in the pathogenesis of SIH, and is mediated by microglia-derived NLRP3 inflammasome activation.

Microglia supervise and respond rapidly to a change of homeostasis, and play the role of immune response by transition of immune phenotype. Stress in the internal or external environment has profound effects on microglial activation. Microglia express different receptors that allow them to respond to stress hormones including glucocorticoids, epinephrine, and norepinephrine, from both peripheral and central sources [41]. Changes in microglia and related hormones might change the activity of neurons in the CNS. During acute and chronic repetitive psychogenic stress, neural activity might change; for example, restraint water-immersion stress alters the neural activity in the central nucleus of the amygdala in rats [42]. The RVLM is the location of pre-sympathetic neurons. It is a structure that integrates multiple descending fibers and projections to sympathetic preganglionic neurons to regulate sympathetic outflow [43, 44], and our previous study demonstrated that microglia in RVLM of SIH rats are activated, and this contributed to the neuroinflammation in SIH [6].

Stress can activate the local RAS in the brain. The critical importance of brain RAS in regulating sympathetic activity and BP is an emerging field of research [45]. It has been reported that bilateral microinjection of human prorenin into the PVN significantly increases splanchnic sympathetic nerve activity [15]. Shi *et al.* [13] demonstrated that prorenin, the precursor of renin in the RAS component, directly activates hypothalamic microglia *in vitro*, showing that RAS-induced neurogenic hypertension is independent of Ang II. Huber *et al.* found that prorenin incubation with brain neurons dramatically enhances the ROS-AP-1-iNOS signaling pathway; they also suggested that PRR activation in the PVN exerts a sympathoexcitation effect. They provided alternative perspectives on the Ang II-independent effect of local RAS [15]. Given the presence of the blood–brain barrier, brain RAS activity depends on the local synthesis of (pro)renin in the brain rather than uptake from blood.

It has been suggested that the brain synthesizes large quantities of prorenin, the inactive precursor of renin [9]. In the brain, it is assumed that prorenin binds to the PRR, which results in prosegment unfolding, thus allowing Ang-I-generating activity without prosegment removal (non-proteolytic activation) [10, 11]. Contrepas *et al.* [46] using *in situ* hybridization showed a wide distribution of PRR mRNA in adult mouse brain, including the RVLM, PVN, supraoptic nucleus, nucleus of the tractus solitarius, and subfornical organ critical for their involvement in the central regulation of cardiovascular function and fluid volume homeostasis. Although neither our studies nor those of others can conclude that pro(renin) is generated in the CNS, they support the hypothesis that pro(renin) plays roles in the brain.

Takahashi *et al.* [47] using immunocytochemistry showed that (P)RR protein is expressed in the paraventricular and supraoptic nuclei of the human hypothalamus, and in anterior pituitary cells of the human brain. Evidence for the presence of PRR in the human brain, both in neurons and microglia, and its positive correlation with SBP provides a foundation for future functional studies on brain PRR in human hypertension. Functionally, it has been demonstrated that PRR antagonists are effective in animal models of hypertension [10]. Furthermore, the expression of PRR in neurons and microglia suggests its possible role in regulating not only neuronal activity but also inflammation and hypertension, a new research avenue in humans [48].

Previous investigators have demonstrated that prorenin and PRR are involved in the pathogenesis of renal and cardiac hypertrophy, accompanied by local oxidative stress, inflammation, and NLRP3-IL-1β-related signals [48, 49]. In chronic inflammation, NLRP3 acts as the primary activator of inflammation, which can trigger the cascade production of other pro-inflammatory cytokines [50, 51]. In salt-induced pre-hypertensive rats, NLRP3 inflammasome activation is associated with hypertension in the PVN [36, 52]. We found that prorenin increased ROS production and triggered microglial M1 phenotype-switching and NLRP3 activation in primary cultured microglia. Furthermore, NLRP3 with mito-tacker staining showed that the translocation of NLRP3 from mitochondria to the cytoplasm was increased in prorenin-challenged microglia. These effects were abolished by co-treatment with the ROS scavenger NAC, an anti-oxidant and anti-inflammatory agent [53]. It should be noted that NAC directly scavenges ROS *via* its sulfhydryl active group; in addition, NAC acts as a glutathione precursor which induces production of the endogenous antioxidant glutathione, thereby reducing the formation of mitochondrial reactive nitrogen species (mROS) [54]. So, NAC can ameliorate mitochondrial dysfunction and decrease oxidative stress in microglia. Ultrastructural microscopic imaging showed swollen mitochondria with disorientated and broken cristae in prorenin-stimulated microglia, while NAC attenuated this injury (Fig. S5). However, the relationship between mROS and NLRP3 activation requires further investigation.

**Fig. 9:**
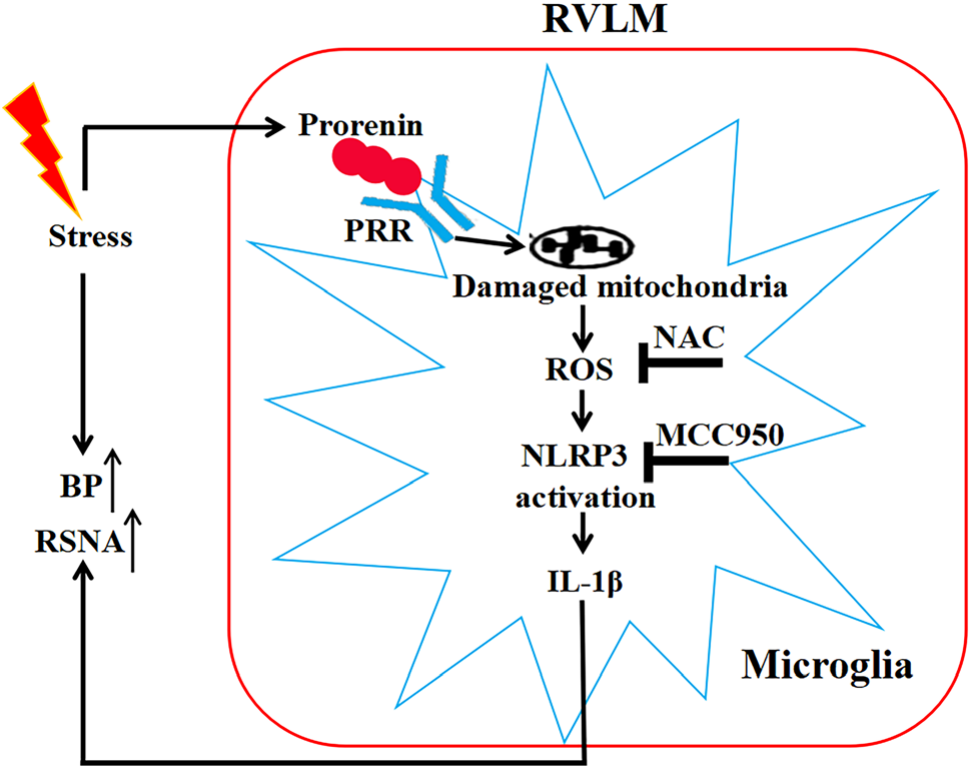
Schematic diagram illustrating the putative mechanisms of the pressor effect of prorenin *via* ROS-triggered microglia-derived NLRP3-IL-1β activation in the RVLM. PRR, (pro)renin receptor; ROS, reactive oxygen species; NAC, N-acetylcysteine; NLRP3, NLR family pyrin domain containing 3; ASC, apoptosis-associated speck-like protein containing a caspase recruitment domain; IL-1β, interleukin-1β; BP, blood pressure; RSNA, renal sympathetic nerve activity.

In summary, external environmental stress upregulates prorenin and PRR expression in the RVLM of stress-exposed rats. ROS overproduction from injured mitochondria induces activation of the microglial NLRP3 inflammasome, after which much more inflammatory factors are released. The neuroinflammation couples with the increased sympathetic flow to underline the pathogeneses of SIH. Blockade of the prorenin-ROS-NLRP3 pathway attenuates this effect (Fig. 9).

## Electronic supplementary material

Below is the link to the electronic supplementary material.

Supplementary material 1 (PDF 2939 kb)
