# Supplementary material for: Microglia-Derived NLRP3 Activation Mediates the Pressor Effect of Prorenin in the Rostral Ventrolateral Medulla of Stress-Induced Hypertensive Rats
Source: Neurosci Bull. 2020 Apr 3;36(5):475–92. doi: 10.1007/s12264-020-00484-9 (PMC7186257; doi:10.1007/s12264-020-00484-9)
Supplement: Supplementary file 1 — Supplementary material 1 (PDF 2939 kb) [file 12264_2020_484_MOESM1_ESM.pdf]

Supplementary Information

Methods

CCK8 assay

The CCK8 assay (Beyotime, Shanghai, China) was used to determine cell viability according to the manufacturer's protocol. A Multiskan FC plate reader (Thermo Scientific, MA) was used to measure absorbance.

RVLM Collection

The medulla oblongata was collected as we described previously [1]. Briefly, after decapitation, the brain was removed and frozen immediately on dry ice. Based on the atlas of Watson and Paxinos, the medulla oblongata containing the RVLM was located between 0.5 and 1.5 mm rostral to the obex, which served as the anatomical landmark. Tissue 1.5 to 2.5 mm lateral to the midline and medial to the spinal trigeminal tract were collected using micropunches.

Transmission Electron microscopy

The procedures were as previously described [2]. Briefly, cultured cells were fixed in Karnovsky's fixative (2% paraformaldehyde with 2.5% glutaraldehyde in 0.1 M phosphate-buffered (PB), pH 7.2–7.4) for 1–2 h at room temperature and then overnight at 4–10°C. The cells were then osmicated, rinsed in phosphate buffer, and dehydrated, then embedded in epoxy resin to polymerize for 24 h at 70°C. Blocks containing microglia were sectioned using an ultramicrotome (Ultracut; Leica) at 70–80 nm. Thin sections were placed on grids and stained with uranyl acetate and

lead citrate. The grids were observed in a transmission electron microscope (H-700; Hitachi, Tokyo, Japan) at 80 kV.

Table S1. Primer sequences

Gene name	Primer sequence	
	Forward	Reverse
Prorenin	5'-TCTTGTTGCTCTGGACCTCTT-3'	5'-GTAATGGTCCCGTTGAAAGT-3'
PRR	5'-CTTTCTGGTGGCGGGTGCTT-3'	5'-ACAGGAGAACGACCAGCACA-3'
NLRP3	5'-TTTCTGACCCTCGTAGTCTATCCA-3'	5'-GGCTGTGGACAATGGGAAGG-3'
ASC	5'-CCCCATAGACCTCACTGATAAA-3'	5'-GCCCATAGCCTTCCCGCACT-3'
Pro-Caspase-1	5'-CTTCTTTGTTCTGCGTCTGC-3'	5'-ATTTGTCCCTTAGAAACAGC-3'
IL-1β	5'-ATGTGCTGCTGCGAGATTTG-3'	5'-CTCAACTGTGAAATGCCACC-3'
TNF-α	5'-GGAAAGCATGATCCGAGATG-3'	5'-CAGTAGACAGAAGAGCGTGGTG-3'
IL-10	5'-CTGCTATGTTGCCTGCTCTT-3'	5'-GGTCAGTCGGTCTGGGTGTA-3'
TGF-β	5'-ATGCCGCCCTCGGGGCTGCGGCTGC-3'	5'-TACGGCGGGAGCCCCGACGCCGACG-3'
β-actin	5'-ACAGCTTCTTTGCAGCTCCTTCG-3'	5'-ATCGTCATCCATGGCGAACTGGTG-3'

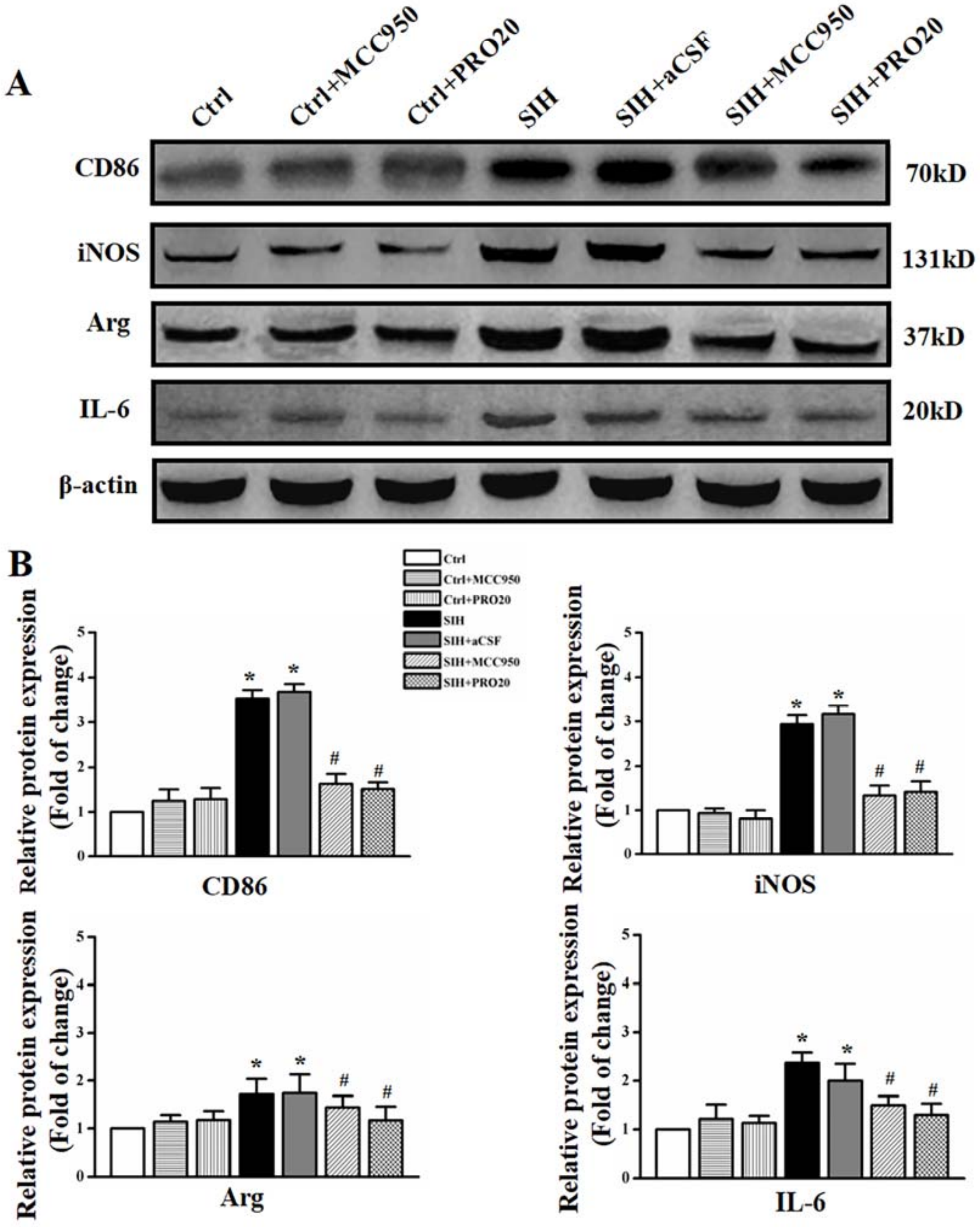

Fig. S1 Effects of MCC950 and PRO20 on M1 polarization in the RVLM of different groups of rats. **A** Representative immunoblots of the expression of the M1 marker CD86 and the main protein products (iNOS, Arg, and IL-6). **B** Densitometric analysis of blots as in **A**. Data represent the mean ± SEM; n = 6; $^{*}P < 0.05$ vs Ctrl; $^{\#}P < 0.05$ *vs* SIH.

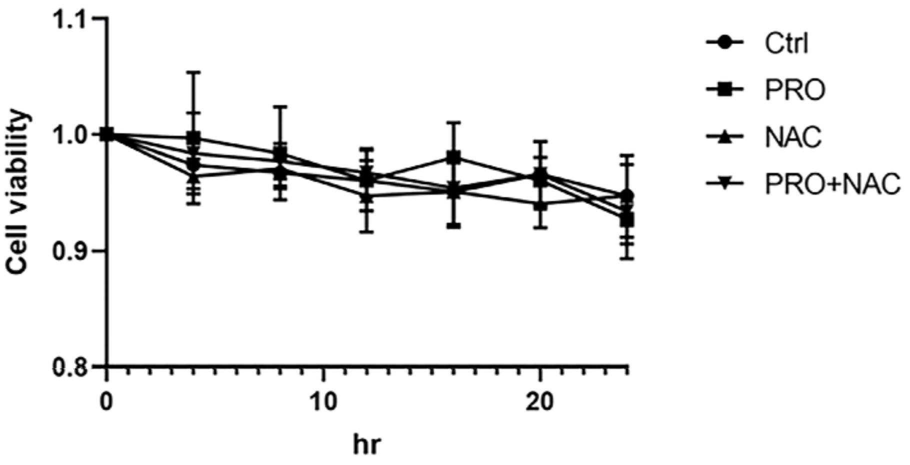

Fig. S2 Viability of microglia in control, prorenin, NAC, and prorenin with NAC groups evaluated using cell counting kit-8 assays as relative 450-nm absorption in different groups. There was no significant difference in viability among the different groups (n = 6).

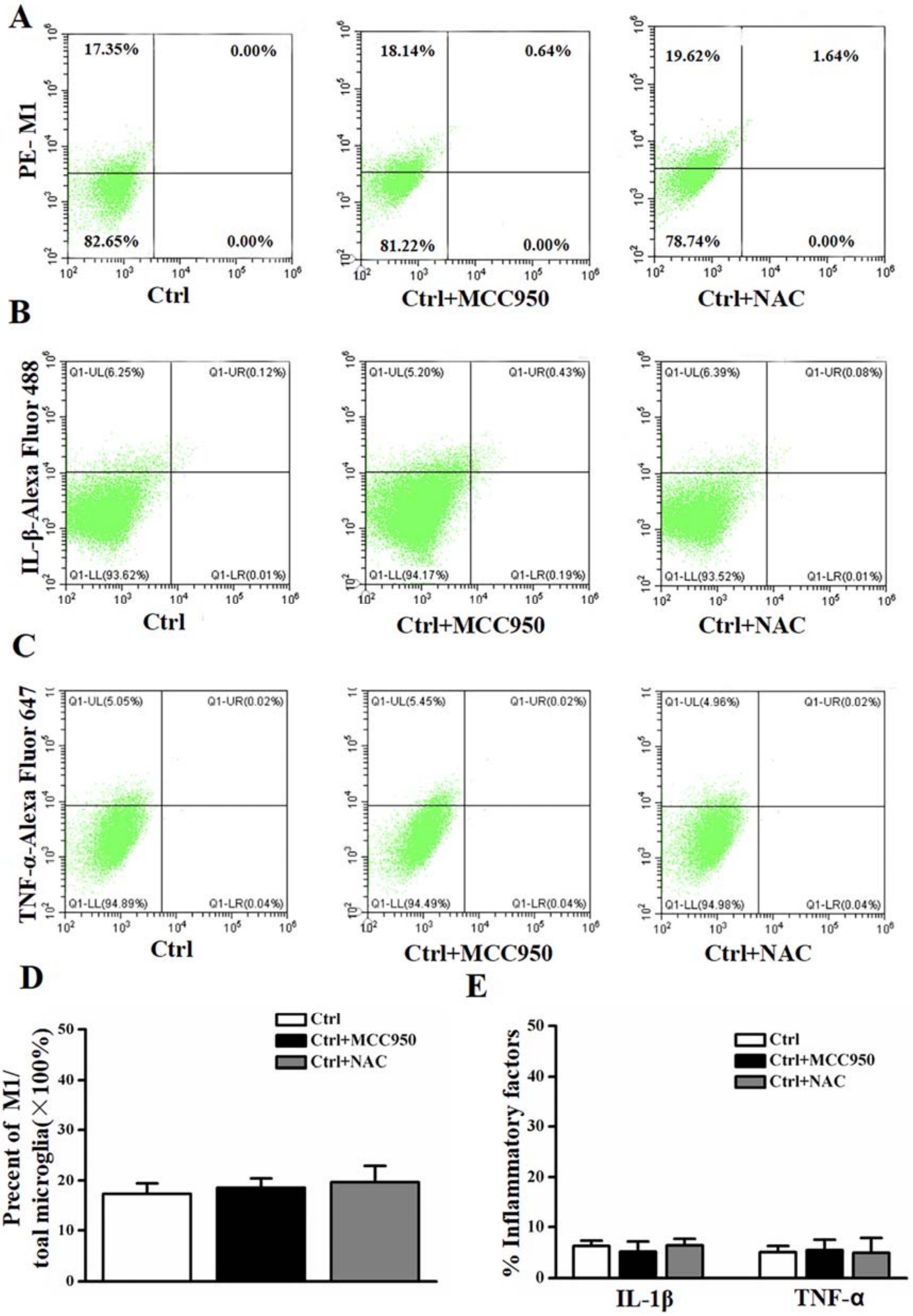

Fig. S3 Effects of MCC950 and NAC on M1 polarization and pro-inflammatory factors in different treated microglia. **A** The M1 phenotype assessed using flow cytometry in Ctrl, Ctrl + MCC950, and Ctrl + NAC-treated microglia. **B, C** The

pro-inflammatory factors IL-1β, TNF-α as assessed by flow cytometry. **D, E** Quantification of M1 phenotype, IL-1β^{+}, and TNF-α^{+} cells in different drug-treated microglia using flow cytometry. Data represent the mean ± SEM (n = 6).

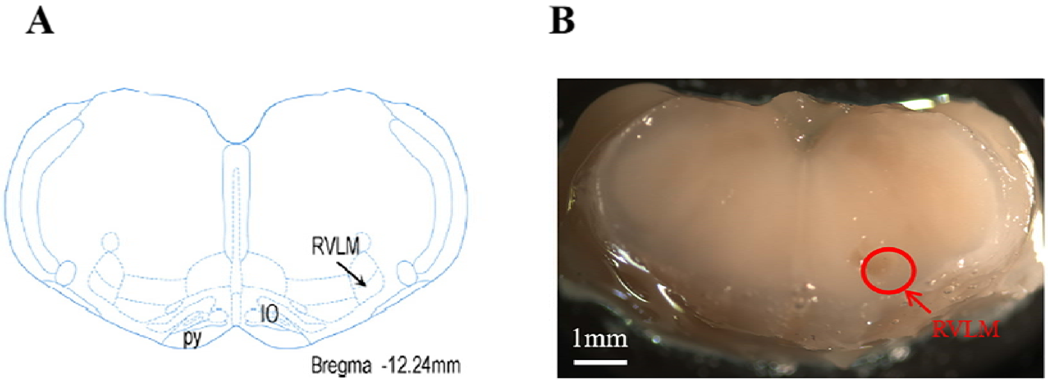

Paxinos G, Watson CR, Emson PC. *J Neurosci Methods* 1980, 3:129-149.

Fig. S4 Identification of RVLM dissection sites. **A, B** Schematic and representative photomicrograph, respectively. The black arrow in **A** and the red arrow in **B** indicate the dissection site. Scale bar, 1 mm in **B**.

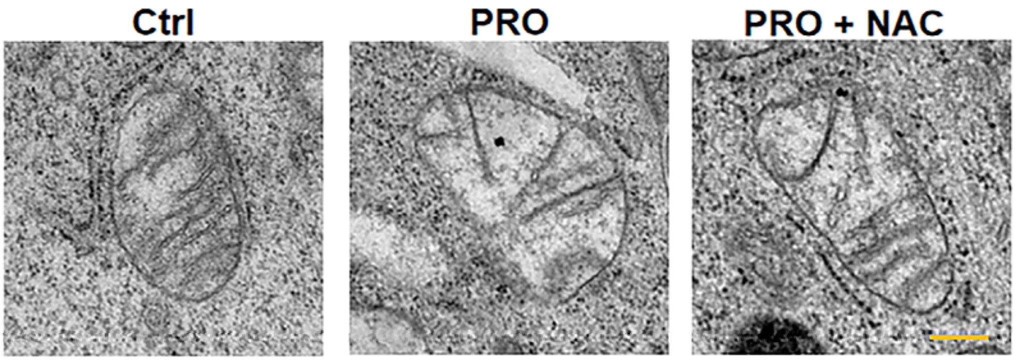

Fig. S5 Representative transmission electron micrographs showing the ultrastructural mitochondrial morphology in microglia. Left panel, normal ultrastructure; middle panel, a swollen mitochondrion with disoriented and broken cristae in a

prorenin-stimulated microglia; right panel, decreased mitochondria injury via inhibition of ROS production Scale bar, 1 nm.

References

[1] Du D, Hu L, Wu J, Wu Q, Cheng W, Guo Y, et al. Neuroinflammation contributes to autophagy flux blockage in the neurons of rostral ventrolateral medulla in stress-induced hypertension rats. J Neuroinflammation 2017,14:169.

[2] Paxinos G, Watson CR, Emson PC. AChE-stained horizontal sections of the rat brain in stereotaxic coordinates. J Neurosci Methods 1980, 3:129-149.

Abbreviations

ROS, reactive oxygen species; RVLM, rostral ventrolateral medulla; SIH, stress-induced hypertension; RAS, renin-angiotensin-system; PVN, paraventricular nucleus. aCSF, artificial cerebrospinal fluid; MAP, mean arterial pressure; RSNA, renal sympathetic nerve activity; PRR, (pro)renin receptor; PIC, proinflammatory cytokine; IL-1β, interleukin-1β; NLRP3, nucleotide-binding oligomerization domain-like receptor containing pyrin domain 3; SBP, systolic blood pressure; Ang II, Angiotensin II; ASC, apoptosis-associated speck-like protein containing a caspase recruitment domain; HR, heart rate; NAC, N-acetylcysteine; CNS, central nervous system.
